# A Case from Practice

**Published:** 1882-04

**Authors:** C. F. Millspaugh

**Affiliations:** Binghamton, N. Y.


### A CASE FROM PRACTICE.

C. F MILLSPAUGH, M. D., BINGHAMTON, N. Y.

MRS. C——, aged thirty-seven, blonde, primipara. I was called to attend in confinement, at 5 A. M., September 5th, and found her in the first stage; the pains having commenced the evening before, at 9 o'clock. She had suffered a great deal of abdominal, hip, thigh and lumbar pain for the last six weeks. I called again at 10.30 A. M., and found the pains very severe, and the expansion of the parts slow, being rigid, the results very inefficient, the pains not accomplishing their object, and the patient very nervous. I gave one dose *Gels.* 30th; from that moment everything progressed nicely until the head reached the perineum; here, the vaginal opening being very tense and constricted, the pain was severe; still, by careful management, and three very strong travails, the child was born into the world, and, much to my relief, without any lacerations of the parts.

It was fifteen minutes after expulsion before the uterus contracted sufficiently to stop the pulsation of the cord. Then, after giving the little 8¼ boy to be washed, I found the placenta already nearly free from the vaginal fissure, and exceedingly large. Judge of my astonishment, when, on attempting to carefully remove this mass, I found that part was still retained; I tried gentle and continuous traction for fifteen minutes at a time, three separate times, for an hour; then, as there was no important hemorrhage and the uterus was well contracted, I gave the patient two hours' rest; during which she slept a few short naps. Upon inserting my hand I found an attachment which was very firm, requiring a half-hour of excruciating pain before it was detached; after detachment I gave one dose *Arn.* 30th, and left.

It is the following prescription that calls forth this article, as I am a true follower of the strict principles which guide the *homœopathic physician:*

I found at 8 o'clock, the next morning, that the soreness and pain which this quite hard birth had caused, and for which I gave the *Arn.*, had very materially diminished, and the following condition present: The urine had not yet passed, nor was there any desire nor sensation; the patient was greatly exhausted and very thirsty, complaining of pain and oppression in the chest, with a dry cough, that of course caused severe pain throughout the abdominal viscera. The whole body was hot, and every time the hand touched the skin she complained that it felt as though the part was being pierced with countless red-hot needles. This would at times prevail over the whole body without any touching of the skin. Here was a situation for a homœopath who had sworn never to use the catheter, nor any accessory means until he had given remedies a good chance. I

opened my case of little pills and found *Ars.* 1 x, 3d, 30th, and 200th D. My fingers itched to pick out the pretty little phial of the 3d, but my vow came before me like Hamlet's ghost; it was too good a chance to lose. I gave one dose of five pellets No. 8 *Ars.*, 200th D., and waited. In just ten minutes she asked for the bed-pan, and passed about ten ounces urine. Rapidly after this the pains, cough, oppression of the chest and the skin irritation passed away, and she dropped into a peaceful sleep, from which she awoke feeling, as she expressed it: "Better than before confinement." Now, reader, if you are narrow-minded enough to say there was no curative power in that dose of *Ars.* 200th, and that all the good results would have happened without it, you should stop the practice of Homœopathy and carry hod for the masons that are building the tomb of allopathy.

